# Parent-child relationship measures and pre-post treatment changes for a clinical preschool sample using DC:0-3R

**DOI:** 10.1186/s13034-024-00746-8

**Published:** 2024-05-16

**Authors:** Nicole Elli Ursula Baans, Marius Janßen, Jörg Michael Müller

**Affiliations:** Department of Child and Adolescent Psychiatry, University Hospital Münster, Schmeddingstrasse 50, 48149 Münster, Germany

**Keywords:** Parent-child relationship, PIRGAS, RPCL, Child and maternal psychopathology

## Abstract

**Background:**

To reduce psychopathologies in children, various treatment approaches focus on the parent-child relationship. Disruptions in the parent-child relationship are outlined in the most recently revised versions of the Diagnostic Classification of Mental Health and Developmental Disorders of Infancy and Early Childhood (DC:0-3R/DC:0–5). The measures used to assess the parent-child relationship include the Parent-Infant Relationship Global Assessment Scale (PIRGAS) and the Relationship Problems Checklist (RPCL), which cover, e.g., essential concepts like over- or underinvolvement of the caregiver. However, not much is known about the cross-sectional and predictive value of PIRGAS and RPCL scores at admission to discharge, namely whether changes in these scores are correlated with child and maternal psychopathologies and changes through treatment.

**Methods:**

Based on clinical records of 174 preschool-aged children of the Family Day Hospital, we report related basic descriptive data and changes from admission to discharge for the parent-child relationship, child behaviour, and maternal psychopathology. We used a Pearson correlation or a point-biserial correlation to describe the associations and performed a paired t-test to examine differences before and after measurement.

**Results:**

Our results show overall improvements in our parent-child relationship measures and in child and maternal psychopathology. However, we observed little or no correlation between the parent-child relationship measures and child or maternal psychopathology.

**Conclusions:**

We highlight potential drawbacks and limitations of the two relationship measures used that may explain the results of this study on the associations between the variables assessed. The discussion emphasizes the assessment of DC:0-3R/DC:0–5, which are popular in clinical practice for economic reasons.

## Background

### Parent-child relationship in the clinical context

In 1994, the Diagnostic Classification of Mental Health and Developmental Disorders of Infancy and Early Childhood (DC:0–3) [1] was introduced to provide an alternative and probably more adequate classification of mental diseases for children between zero and three years of age [2]. This manual was later revised (DC:0-3R) [3] and then extended to preschool age with the DC:0–5 [4, 5]. In the DC:0-3R manual, a diagnostic framework is given to assess the disorder in a parent-child relationship (PCR) (DC 0–3: Axis II), and two measures are introduced: The Parent-Infant Relationship Global Assessment Scale (PIRGAS) and the Relationship Problems Checklist (RPCL) [1, 3]. The PIRGAS is a global one-item measure with a scale from 0 to 100, where scores between 91 and 100 label a relationship as *well adapted*, 81–90 as *adapted*, 71–80 as *perturbed*, 61–70 as *significantly perturbed*, 51–60 as *distressed*, 41–50 as *disturbed*, 31–40 as *disordered*, 21–30 as *severely disordered*, 11–20 as *grossly impaired*, and 1–10 as *documented maltreatment* [3]. Additionally, according to the manual, each dyad’s relationship is labelled as a *disturbed relationship* if the PIRGAS ≤ 40 [3]. Please note that the wording and number expression is given in the manual and does not represent our interpretation.

The RPCL classifies relevant parental behaviour for relationship problems using seven global one-item measures, labelling parenting behaviour as *overinvolved*, *underinvolved*, *anxious/tense*, *angry/hostile*, *verbally abusive, physically abusive*, or *sexually abusive* [3].

The PIRGAS and RPCL are potential candidates for assessing important theoretical and practical information to explain and treat child psychopathology [6]—even though they are more subjective and therefore susceptible to the biases inherent to all clinical assessments [7]—because they can be applied easily and quickly; this is particularly important in times of increasing economic pressure. This makes global assessment approaches more attractive than multiple-item questionnaires or observational instruments that require training. Also, from a validity perspective, a global measure may document clinical impressions better by taking the unique circumstances of a parent-child relationship into account.

However, a disadvantage of these measures is that the assessment approach in PIRGAS and RPCL is not standardized in terms of recommendations on training or regarding how long or in what setting a parent-child dyad should be observed to obtain reliable clinical information [8, 9]. While the DC:0–3/0-3R does provide vague diagnostic guidelines and names aspects that should be included in a full diagnostic evaluation [1, 3], the manual does not provide any references to a clear theoretical background or related empirical studies. A potential further limitation is that the RPCL provides only a dichotomous classification instead of a graded dimensional score. Additionally, the RPCL domains represent less a direct measure of relationship quality and instead focus on parental behaviour as cause or reaction within a reciprocal interaction schema which is also bidirectionally affected by parental and child distress [10–12]. Thus, our paper does not intend to add or clarify the scientific basis for these measures. Instead, given the widespread use and attractiveness of these measures, we want to know whether these global measures are useful in the context of daily routine diagnostics. In our study, *useful* means that the measures show covariation and concordant changes to child and maternal psychopathology. In the following section, we describe the broad usage of both measures in Table 1. Note that because the newer version DC:0–5 published in 2016 lacks instruments for assessing the parent-child relationship, we assume that the PIRGAS and RPCL may still be applied in clinical practice or still used in research studies, as in Brann et al., 2021 [13].

### Pirgas and the Rpcl

PIRGAS and the RPCL show a wide range between study samples (Table 1). Skovgaard et al. indicated that the methodological diversity between the studies may explain the large variation in frequencies [14], but it may also be due to the largely unstandardized assessment conditions regarding observed interaction settings, raters, and duration of the observation as well as the absence of clear criteria for assigning the diagnosis on Axis II [9].

**Table 1:** Frequency distribution of the RPCL subgroups from six studies

Study	*N*	No rel. diagnosis	Over- involved	Under- involved	Anxious tensed	Angry hostile	Abusive
Cordeiro et al., 2003* [15]	343	13.12%	4.37%	29.45%	9.62%	2.92%	3.21%
Keren et al., 2003* [16]	414	48.0%	11.1%	4.7%	12.1%	3.0%	0.7%
Minde & Tidmarsh, 1997* [17]	57	47.37%	10.53%	35.09%	5.36%	1.75%	
Maldonado-Durán et al., 2003* [18]	167	62.8%	7.18%	22.75%	2.3%	2.3%	0.5%
Akca et al., 2012* [19]	457	25.2%	3.5%	52.1%	4.8%	3.5%	12.0%
Skovgaard et al., 2007** [20]	211			5.2%	0.5%	0.5%	

*Clinical sample, **random sample

An association between PIRGAS and RPCL measures and specific diagnoses according to Axis I of the DC:0–3/0-3R could have not be found [18], but an association does exist on a more global level of having or not having any mental health diagnosis according to ICD 10 [20, 21]. The PIRGAS shows a spearman correlation of *r* = −.23 with aggressiveness [22], a correlation with increased internalizing behaviour [23], and moderate [24] to strong effects [13] in treatment evaluation. Therefore, the clinical value of the RPCL categories may be in their ability to point to parental behaviours that may explain disruptions in the parent-child relationship; these behaviours may lead to specific treatment goals and therapeutic foci. In the preprint of this article, we described several correlations regarding parenting behaviours that can lead to increased internalizing or externalizing problems [25].

### Research question

Before describing our research question, it is important to describe the general treatment effects we observed in terms of the impact on child psychopathology [26] and in relation to parental outcomes [27]. Specifically, we observed an improvement in child psychopathology (d = -0.50) and parental psychopathology (d = 1.64) [26, 27]. Given these effects, we would expect concordant changes in parenting behaviour described by the RPCL and PIRGAS.

## Methods

### The Family Day Hospital

The Family Day Hospital is a part of the Clinic for Child and Adolescent Psychiatry, Psychosomatics, and Psychotherapy of the University Hospital in Münster, Germany, and provides an eclectic interactional family-centred approach for infants, toddlers, and preschoolers from birth to approximately six years of age and their caregivers as part of a multimodal approach [28, 29]. The treatments include parent groups, children’s groups, video-based parent-child interaction therapy, and individual sessions with the parents and family [28].

## Procedure

Our data are based on a retrospective clinical record data collection for 174 children and their caregivers treated at the Family Day Hospital between 2002 and 2012. This study was approved by the local ethics committee of the University Hospital of Münster.

## Sample

The characteristics of the sample are presented in Table 2. Most dyads in our sample consisted of mother and child (171 out of 174).

**Table 2:** Description of the sample and the therapy (*N* = 174)

	Variable		No. (%) or M (SD)
Children	Gender	Female	52 (29.9%)
		Male	122 (70.1%)
	Age (years)	Range 0;4–7;10	4.65 (1.48)
	Nationality	German	171 (98.3%)
	Living with	Both parents	130 (74.7%)
		One parent	40 (74.7%)
		Others	4 (2.3%)
	Children in the household	1	67 (38.5%)
		2	81 (46.6%)
		> 2	26 (14.9%)
Mothers	Age (years)		33.40 (6.44)
	Nationality	German	152 (87.4%)
	Educational status	Secondary school certificate	101 (58.0%)
		A-levels	31 (17.8%)
		Other	42 (24.2%)
	Marital status	Married	115 (66.1%)
		Single	31 17.8%)
		Others	28 (16.1%)
Therapy	Duration (months)		4.27 (2.11)

## Measures

PIRGAS and the RPCL were already introduced in the introduction. It should be noted that the frequency of all ‘abusive’ RPCL categories was very low or zero, so these were not included in the subsequent analysis. Furthermore, therapists and parents filled out the TRF/CBCL 1.5-5 as described in Müller et al. 2011 and 2013 [30, 31]. Their composite score was used to facilitate the analysis. Moreover, the Symptom Checklist 90-Revised (SCL-90-R) [32] was completed by the same parent.

## Statistical analysis

We performed the statistical analysis using the Statistical Package for Social Science (IMB SPSS 29). The alpha level was set at *p* <.05, and one-tailed testing was applied whenever reasonable.

## Results

### Parent-child relationship and psychopathology at admission and discharge

Shown in Table 3, the PIRGAS classified 58.38% of the clinical sample as having a disturbed relationship, while 88.24% was classified as having at least one questionable parenting behaviour. The frequency profile across the RPCL subgroup classifications was relatively stable from admission to discharge, but by the end of therapy these frequencies were reduced by 10–20% depending on the RPCL category. The greatest reduction was observed for the category *angry/hostile*.

**Table 3:** Pre- and post-treatment scores and changes in PIRGAS, RPCL, child behaviour, and maternal psychopathology

	Admission	Discharge	Change from admission to discharge			
	Evidence (+) %	Evidence (+) %	+/+ %	+/ − %	− /+ %	− / − %
PIRGAS ≤ 40	58.38	15.03	13.87	44.51	1.16	40.46
*RPCL*						
Overinvolved	26.05	16.81	15.13	10.92*	1.68	75.27
Underinvolved	24.58	15.25	11.02	13.56*	4.24	71.19
Anxious/tense	52.94	44.54	37.82	15.13	6.72	40.34
Angry/hostile	35.29	15.13	13.45	21.85*	1.68	63.03
Verbal abusive	0	0	0	0	0	100
Physical abusive	0.68	0.68	100	0	0	0
Sexual abusive	0	0	0	0	0	100
Any RPCL	88.24	71.43	68.91	19.33	2.52	9.24

		Admission	Discharge			Cohen
		M (SD)	M (SD)	t	p	d
	PIRGAS	45.84 (13.38)	59.08 (13.39)	− 14.44	< 0.001	− 1.10
CBCL/TRF	Internalizing	58.59 (7.73)	53.79 (7.15)	7.78	< 0.001	− 0.64
	Externalizing	59.32 (8.81)	53.54 (7.35)	8.87	< 0.001	− 0.73
SCL	GSI	0.72 (0.51)	0.37 (0.34)	8.34	< 0.001	− 0.84

*p < .05; **p < .01; ***p < .001

Multiple classifications in RPCL are possible. Changes in PIRGAS/RPCL from admission to discharge were tested with McNemar*. Scores for child behaviour were based on an aggregate of maternal (CBCL) and therapeutic ratings (TRF)

### Changes between parent-child relationship and clinical symptom scales

In Table 4 we present the bivariate associations between PIRGAS and RPCL measures with the clinical symptom scale of child externalizing and internalizing behaviour and maternal psychopathology at admission, discharge, and their concordant changes.

**Table 4:** Child and maternal psychopathology and their association to PIRGAS and RPCL

		Child internalizing	Child externalizing	Maternal psychopathology
		Admission		
Admission	PIRGAS	− 0.10	− 0.18*	− 0.13
	Overinvolved	0.06	− 0.12	− 0.02
	Underinvolved	− 0.00	− 0.06	− 0.06
	Anxious/tense	− 0.10	0.07	− 0.04
	Angry/hostile	0.01	0.08	0.15
			Discharge	
Discharge	PIRGAS	− 0.09	− 0.19*	− 0.16
	Overinvolved	0.03	− 0.09	0.14
	Underinvolved	0.06	− 0.11	0.01
	Anxious/tense	− 0.01	0.07	0.16
	Angry/hostile	0.03	0.04	0.31**
			Delta	
Delta	PIRGAS	− 0.27***	− 0.20*	− 0.19
	Overinvolved	− 0.12	0.14	− 0.12
	Underinvolved	0.09	0.08	0.10
	Anxious/tense	0.11	0.09	0.11
	Angry/hostile	− 0.05	0.00	− 0.04

*p < .05; **p < .01; ***p < .001

Delta: Pre-post treatment changes

## Discussion

### Parent-child relationship diagnostic at admission and changes during treatment

The main question of this study relates to whether global assessments of parent-child relationship (PIRGAS) and parental behaviour (RPCL categories) used in routine clinical practice predict a concomitant reduction in child or parental psychopathology when improved. Underpinning this analysis are the previously reported strong improvements related to children’s internalizing and externalizing symptoms as well as parental psychopathology in the course of treatment at the Family Day Hospital [26, 27]. Additionally, in relation to the RPCL, we observed the occurrence of questionable parental behaviour at admission and a reduction at discharge with respect to *overinvolved*, *underinvolved*, and especially *angry/hostile* behaviour; the reduction in *anxious/tense* behaviour was not significant. The PIRGAS cutoff classification indicated a disturbed relationship for approximately 40% of the parent-child dyads at admission, while at discharge the metric PIRGAS score showed a considerable improvement of d = − 1.1. Related to our main hypothesis, we did not observe concordant improvement between the improvements related to the RPCL categories and children’s and parents’ psychopathology and only a weak association between improvement in PIRGAS and children’s internalizing and externalizing behaviour. This pattern was already apparent for the cross-sectional scores at admission and at discharge.

Our findings can be explained by the assumption that the RPCL categories in particular are not sufficiently standardized, which is likely a consequence of single-item global ratings with a dichotomous answer format. This may also explain the variation in prevalence in other studies (Table 1). Aside from the methodological limitations of global ratings, we consider conceptual limitations to be even more important because the RPCL was designed to assess questionable or potential negative parental behaviour but not diverse variants of positive parenting behaviour like scaffolding, support, or accepting behaviour, whose promotion is probably more effective than eliminating dysfunctional behaviours [33]. The DC:0–5 no longer includes these assessment instruments, but because clinicians still consider the content and observational approach as highly relevant to their work [34] and the DC:0–5 lacks comparable alternative measurement instruments, they are likely to still use these measures in practice for diagnostics and to validate other measures, as in Brann et al., 2021 [13].

### Limitations and strengths

Our clinical sample covered a considerable variation and improvement in child and maternal psychopathology as well as in the parent-child relationship measures within a longitudinal design. Moreover, the sample size allowed for sufficient statistical power, and the data were collected with a focus on proximity to real practice during the clinic staff’s daily routines. However, we did not include data belonging to father-child dyads or relationships to other family members who may be important for the child in question. Our results should be interpreted with caution and should not be overrated in terms of their importance for guiding practitioners’ case formulation and treatment planning.

## Conclusions

Global measures such as PIRGAS and RPCL are popular in times of growing economic pressure, not simply due to their ease of use. However, we see many disadvantages concerning the reliability and design of the RPCL, particularly with respect to the lack of assessment of positive parenting behaviour. The lack of association between the RPCL categories and the PIRGAS also indicates that the PIRGAS may not cover all facets of a disturbed relationship. These limitations may also explain why changes in children’s internalizing or externalizing behaviour were not associated with improvements in PIRGAS or reductions in negative parental behaviour (RPCL). We conclude that further test development to assess clinically relevant aspects and constructs and their validation is still needed.

## Data Availability

The datasets used and/or analysed during the current study are available from the corresponding author on reasonable request.

## Abbreviations

DC:0-3R: Diagnostic Classification of Mental Health and Developmental Disorders of Infancy and Early Childhood. Revised edition (DC:0-3R)
DC:0-5: Diagnostic Classification of Mental Health and Developmental Disorders of Infancy and Childhood: Revised edition (DC:0-5)
PCR: Parent-child relationship
PIRGAS: Parent-Infant Relationship Global Assessment Scale
RPCL: Relationship Problems Checklist
TRF: Teacher’s Report Form
SCL-90-R: Symptom Checklist 90-Revised
GSI: Global Severity Index
